# Pandemic Influenza Planning in the United States from a Health Disparities Perspective

**DOI:** 10.3201/eid1405.071301

**Published:** 2008-05

**Authors:** Philip Blumenshine, Arthur Reingold, Susan Egerter, Robin Mockenhaupt, Paula Braveman, James Marks

**Affiliations:** * Weill/Cornell Medical College, Ithaca, New York, USA; † University of California, Berkeley, California, USA; ‡ University of California, San Francisco, California, USA; § Robert Wood Johnson Foundation, Princeton, New Jersey, USA; 1 Current affiliation: University of California, San Francisco, California, USA

**Keywords:** Socioeconomic factors, ethnic groups, influenza A virus, influenza, human epidemiology, disease outbreaks, disaster planning, health services accessibility, perspective

## Abstract

We explored how different socioeconomic and racial/ethnic groups in the United States might fare in an influenza pandemic on the basis of social factors that shape exposure, vulnerability to influenza virus, and timeliness and adequacy of treatment. We discuss policies that might differentially affect social groups’ risk for illness or death. Our purpose is not to establish the precise magnitude of disparities likely to occur; rather, it is to call attention to avoidable disparities that can be expected in the absence of systematic attention to differential social risks in pandemic preparedness plans. Policy makers at the federal, state, and local levels should consider potential sources of socioeconomic and racial/ethnic disparities during a pandemic and formulate specific plans to minimize these disparities.

## Abstract 2

Preparedness plans must account for the fact that illness and death rates may differ for members of some socioeconomic and racial/ethnic groups during a pandemic.

The threat of pandemic influenza has generated concern among politicians, policy makers, healthcare professionals, and the general public. For the past several centuries, major influenza pandemics have occurred every 10 to 30 years (*1*); it is widely believed that a new pandemic is “inevitable” (*2*). The possibility of an imminent influenza pandemic has been heightened by the appearance and spread of avian influenza A (H5N1), which has a case-fatality ratio of >50% (*3*). Although the assumption has been that avian influenza viruses could not directly infect humans, the transmission of influenza virus (H5N1) directly from chickens to humans in 1997 caused experts to reconsider that assumption (*4*). Genetic changes in influenza virus subtype H5N1 in 2003 resulted in a new strain of the virus, which spread to multiple countries in East and Southeast Asia (*5*), as well as Europe and Africa. Whether the avian influenza virus (H5N1) develops human pandemic potential, its spread from birds to humans and the severity of the resulting disease have heightened concerns about a possible future influenza pandemic.

Considerable financial resources have been devoted to pandemic influenza preparedness planning at the federal and state levels (*6**,**7*); however, resources at state and local levels may be inadequate to implement a robust preparedness plan (*8**,**9*). Past experience with natural disasters and current socioeconomic and racial/ethnic disparities in healthcare in the United States (*10**,**11*) raise questions about the adequacy of plans to address the needs of disadvantaged populations. For example, in responding to Hurricane Katrina, planners apparently failed to consider that many low-income persons might lack private modes of transportation and would depend on institutional help for evacuation. Although the evacuation was successful overall (*12*), deaths, injuries, and illness occurred disproportionately among low-income persons in New Orleans because of economic and logistic constraints on their ability to respond to government recommendations to leave the city. Low-income and disadvantaged persons often suffer disproportionately during natural disasters and epidemics, and historical evidence demonstrates that low-income persons fared considerably worse than high-income persons during the 1918 pandemic in the United States (*13*).

In this article, we describe ways in which different socioeconomic and racial/ethnic groups might fare differently in an influenza pandemic, on the basis of current knowledge of social factors that shape exposure and vulnerability to influenza virus and that influence the timeliness and adequacy of treatment among those who become ill. We also discuss policy decisions, made either before or during a pandemic, which might differentially affect risk for illness or death for those of low income and of specific racial/ethnic groups. Our purposes are to 1) call attention to potentially major and avoidable social disparities in suffering and death during an influenza pandemic and 2) highlight the importance of including in pandemic preparedness plans targeted strategies for minimizing or avoiding these social disparities. The following discussion is not meant to be exhaustive; rather, it is meant to provoke reflection about how potential disparities in the effects of an influenza pandemic might be reduced or eliminated through appropriate planning and implementation of clinical and public health activities.

## Conceptual Framework

Using a conceptual framework adapted from Diderichsen et al. (*14*), we systematically considered possible sources of disparities during an influenza pandemic by examining the following 3 levels at which underlying socioeconomic or racial/ethnic differences could lead to disparities in illness or death: 1) likelihood of being exposed to the influenza virus; 2) likelihood of contracting influenza disease, if exposed; and 3) likelihood of receiving timely and effective treatment after influenza disease has developed. To explore socioeconomic and racial/ethnic disparities at each level, we searched the literature for relevant findings based on population-based national data (Figure, Table).

**Figure:**
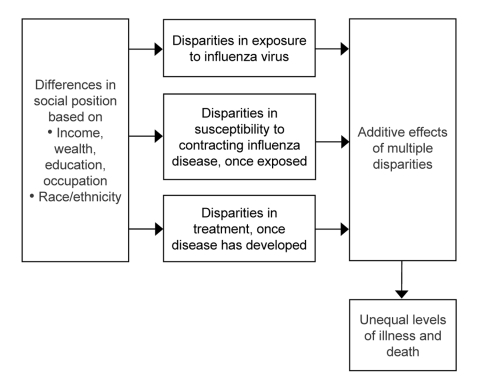
Possible sources of disparities during a pandemic influenza outbreak.

**Table:** Factors that could contribute to health disparities among socioeconomic and racial/ethnic groups during an influenza pandemic

Differences in exposure to influenza virus
Crowding in households, medical facilities, public transportation
Occupational factors such as inability to work from home, dependence on childcare outside of the home
Differences in susceptibility to influenza disease, once exposed to the virus
Host factors, including preexisting immunity, age, other underlying diseases or conditions, smoking, nutritional status, stress
Vaccination status, reflecting differences in vaccine seeking and acceptance and in vaccine availability
Differences in timely effective treatment, once influenza disease has developed
Access to outpatient and inpatient medical care
Care-seeking attitudes and behavior
Financial obstacles, including lack of adequate insurance coverage
Logistic obstacles, including transportation, language
Quality of care
Availability of antiviral treatments
Appropriate inpatient treatment

## How Could Disparities Arise?

### Differences in Exposure

Regardless of which strain of influenza virus causes the next pandemic, it will be highly transmissible between humans. Transmission of influenza is primarily airborne, through aerosolized respiratory tract secretions expelled during coughing and sneezing, although transmission by direct and possibly indirect contact may occur. Transmission can be expected to occur in various settings, including homes, healthcare facilities, schools, work sites, public transportation, and other settings at which people gather for social, commercial, or entertainment purposes. Higher exposure risk among particular population groups as a result of factors such as crowding and occupation could contribute to health disparities among socioeconomic and racial/ethnic groups during an influenza pandemic.

Crowding, an established risk factor for many infectious diseases, can increase the likelihood of pathogen transmission. In the United States, urban poverty and Hispanic and Asian ethnicity are correlated with domestic crowding; even at higher income levels, Hispanic and Asian households are relatively more crowded than white and African-American households (*15*). In addition, in the United States, low-income persons, African Americans, and nonwhite Hispanics are more likely than persons in other groups to obtain regular medical care at emergency departments and publicly funded clinics (*10*), where airborne transmission of infectious agents has been documented. Because these locations typically do not segregate sick and well patients and are becoming increasingly crowded (*16*), patients waiting for care in these settings are likely to have greater exposure to influenza viruses and other respiratory pathogens. Another source of increased exposure to infected persons is public transportation, where persons from low-income and minority households account for 63% of users (*17*).

Occupational factors are also likely to lead to differential exposure risk during an influenza pandemic, particularly in terms of adherence to strategies that aim to limit case-patient contact with others (*18*). Staying home may not be economically feasible for persons in lower wage occupations; these persons are less able to afford losing income as a result of missed work and often lack the job flexibility that would permit them to work at home. In addition, their jobs may be necessary because they provide essential goods and services. For these reasons, parents in lower wage/lower status occupations may be more likely to keep their children in communal childcare settings—where exposure risks are relatively high—during an influenza pandemic, placing everyone in the family at greater risk for exposure.

### Differences in Susceptibility

Among persons who have been exposed to influenza virus, the likelihood of contracting disease may be modified by underlying host factors and medical conditions, such as age, smoking status, nutritional status, stress levels, and cardiopulmonary disease. The influence that most host factors will have on the development of influenza during a future pandemic is uncertain; some evidence suggests that the factors affecting disease severity and death may differ from those typically observed during annual influenza epidemics (*19*). However, given overwhelming evidence that low-income persons are generally more susceptible to infectious diseases, it is reasonable to plan on the basis of well-documented annual epidemic patterns, in which influenza disease development is influenced by factors that are differentially distributed across socioeconomic and racial/ethnic groups. These patterns, as well as patterns of many other diseases, indicate that socially disadvantaged groups are likely to be at higher risk for influenza disease, particularly severe disease.

The inability to predict which influenza virus will cause a future pandemic, together with the very limited national and global capacity to produce influenza vaccine in massive quantities in a short time, almost ensures that an effective vaccine will be unavailable to most or all of the population during the early stages of a pandemic and in very short supply thereafter. Even so, current plans assume that local and state public health agencies will have a primary role for distributing pandemic influenza vaccine. In general, however, these plans do not adequately address preventing or minimizing socioeconomic or racial/ethnic disparities in vaccine distribution and acceptance, despite evidence that such disparities have been the rule for the annual influenza vaccine, even among persons >65 years of age (*20*). In the United States, routine use of annual influenza vaccine in preschool children has only recently been introduced; information focusing on school-age children is limited (*21*). Nevertheless, African American/black children and children from lower income families, who are at higher risk of contracting influenza (*22*) in this country, are less likely to be up to date with other routine immunizations (*23*). It is possible that, in the context of an influenza pandemic, vaccine-seeking and acceptance behavior and resultant coverage patterns may differ from those observed during routine vaccination efforts; however, the weight of available evidence indicates that social disparities in vaccine coverage are likely to occur in the absence of careful planning to prevent them.

### Differences in Treatment

Among those who contract influenza, subsequent illness and death may be influenced by underlying factors and conditions and by the timeliness and effectiveness of various treatment modalities. Most influenza illnesses are self-limiting, and most infected persons during both annual influenza epidemics and influenza pandemics (including that of 1918–19) recover with only supportive care in the community. Even so, current planning efforts recognize the potential importance of reducing disease during a pandemic, through early treatment with antiviral drugs and through other forms of treatment such as respiratory support and antimicrobial agents to treat secondary bacterial pneumonia, among those with more severe disease.

In the United States, the likelihood of substantial disparities in access to timely and appropriate care under influenza pandemic conditions is high, given long-standing and persistent disparities in access to medical care. For example, persons with low income are ≈2× as likely as those with higher incomes to lack a usual source of healthcare (*24*). Similarly, non-Hispanic black and Hispanic persons are significantly less likely than non-Hispanic white persons to report having a usual primary care provider (*10*). Among persons who do report having a usual source of care, those who are poor or near poor and those who are non-Hispanic black or Hispanic are 2.5–4× as likely as their relatively higher income and white counterparts to rely on a hospital-based source of primary care (*24*). These same groups are also more likely to report having difficulty obtaining timely appointments for illness or injury, which suggests problems with access to care even among those with a usual source of healthcare (*10*). Language and cultural barriers to seeking and receiving medical care also may contribute to disparities. In emergency departments, for example, interpreters are frequently unavailable or underused, which has potentially adverse implications for patients’ understanding of their disease or treatment and for clinical decision making and quality of care (*25*). In addition, the large numbers of persons who lack health insurance, as well as those who lack documentation of US citizenship, often delay seeking care because they are concerned about paying for the care or encountering legal difficulties.

Evidence from previous outbreaks suggests that antiviral drugs may be effective for treatment (*26*) and prevention (*27*) of pandemic influenza, and current antiviral drugs seem to be biologically effective against 1918 and 1918-like viruses (*28*). Because vaccine may not be available when a pandemic begins, experts have suggested that the antiviral drug oseltamivir should be stockpiled for use during a pandemic influenza outbreak. Recent models suggest that early use of oseltamivir may contain outbreaks if certain criteria regarding transmissibility and compliance are met (*29*). However, experience with nonpandemic influenza indicates that oseltamivir must be given early during symptom development for it to have any substantial biological effect (*30*); modest delays may vitiate the treatment effectiveness (*31*). Although plans for release and distribution of antiviral drugs are still being finalized, overcoming long-standing disparities in access to timely treatment by socioeconomic status, race/ethnicity, ability to speak English, and legal status will present numerous challenges to ensuring equal access to such drugs during a pandemic.

Reasons for concern about disparities in the timeliness and appropriateness of the care received by influenza patients who might benefit from in-hospital care are similar. Given the predicted insufficient supply of hospital beds and staff during a pandemic (*32*), a person’s access to potentially lifesaving therapies such as respiratory support and antimicrobial treatment of secondary bacterial pneumonias in an inpatient setting is likely to depend on factors that include usual source of care, citizenship status, and ability to speak English. Disparities may also occur in the quality of care received by persons who are hospitalized. Earlier US studies of persons hospitalized for pneumonia have found that blacks and “other minorities” are 71% and 79% as likely, respectively, as non-Hispanic whites to receive antimicrobial agents within 8 hours of arrival at the hospital (*33*) and significantly less likely to have blood cultures obtained before receiving antimicrobial therapy (*10*). Such disparities in quality of care would likely persist during an influenza pandemic.

## Discussion

Although reducing or eliminating socioeconomic and racial/ethnic disparities in health and healthcare has been an official federal and state policy priority for 2 decades (*34*), such disparities remain prevalent and may inadvertently become wider when not explicitly addressed by policies designed to improve the health of the population as a whole and of disadvantaged persons in particular (*35*). Given the current limitations of our public health infrastructure and the disparities in healthcare, a pandemic influenza outbreak in the United States is likely to disproportionately affect persons from socially disadvantaged groups. Explicit, systematic, and detailed plans are essential for overcoming the social barriers that are predicted to result in socioeconomic and racial/ethnic disparities in pandemic influenza illness and death. Saunders and Monet also have called for pandemic influenza planning that appropriately considers the needs of disadvantaged populations (*36*).

The Pandemic Influenza Plan of the US Department of Health and Human Services (HHS) (*37*) does not adequately address potential social disparities in exposure, vaccination, or treatment; the possible effects of such disparities; or strategies for minimizing or eliminating them. The HHS plan (*37*), the federal guidance on vaccine allocation (*38*), and the recent Centers for Disease Control and Prevention (CDC) guidelines for community-level mitigation strategies (*18*) should be credited for calling for community engagement and inclusion of a wide variety of stakeholders in planning at the local level. Outreach to providers, community leaders, and organizations, particularly in disadvantaged communities, will be an important component of any strategy for addressing disparities during a pandemic. However, the available versions of official plans do not call attention to the need for special efforts to overcome the greater barriers likely to be faced by socially disadvantaged groups.

On a US government website for pandemic influenza (www.pandemicflu.gov), a question asks which groups would be especially vulnerable during an influenza pandemic. The answer notes that people may be vulnerable for a variety of reasons, including limited access to healthcare; limited proficiency in English; or being disabled, homeless, economically disadvantaged, or a single parent. The response calls for faith-based and community-based organizations to develop plans “to care for dependent populations” and to “provide financial aid to the poor who are unable to work and are in need of emergency income for housing, medicine, or other essential needs” (www.pandemicflu.gov/faq/pandemicinfluenza/pi-0001.html), which implies that attention to the needs of economically or socially vulnerable persons is not primarily a public-sector responsibility but is more a matter for private charity. The 2005 HHS plan (*37*) itself acknowledges that some groups may need financial assistance if they are unable to work but does not indicate how that assistance would be provided or who would provide it.

Those who are still formulating plans should consider likely differences in influenza exposure and identify potential strategies for mitigating such disparities. Mathematical models have demonstrated that community-based interventions, such as quarantine and individual isolation, may be important for reducing influenza attack rates and overall incidence (*29*). Most pandemic plans call for limiting public gatherings and closing schools to slow the spread of influenza, without adequately taking into account how implementing these strategies could differentially affect disadvantaged groups. Recent recommendations from CDC go further in recognizing the differential effect of social-distancing measures on vulnerable communities (*18*). Although CDC advocates flexible work arrangements, income replacement, and job security to minimize the negative effects of social-distancing measures, it pays inadequate attention to those whose jobs will not accommodate these interventions. More specific solutions should be outlined in pandemic preparedness plans to address the economic effects of quarantine on low-income persons, who by staying home may be at risk wage loss, job termination, or both. Job security and income replacement are key components to limiting the effects of potential quarantine measures on disadvantaged persons (*39*) and should be extended to all persons, regardless of their type of work.

Important decisions also will need to be made concerning access to vaccination and treatment in the event of a pandemic. The federal government’s Draft Guidance on Allocating and Targeting Pandemic Influenza Vaccine (*38*) provides a basic framework for allocating vaccine during the pandemic. An appendix to that document mentions (on p.17) that the principles of “fairness and equity (recognizing that all persons have equal value, and providing equal opportunity for vaccination among all persons in a priority group)” were considered when drafting the guidelines. Although the proposed schema very reasonably first defines groups of different priority levels according to occupation and then, within the general population, according to age and pregnancy status, it does not provide explicit attention to groups who are vulnerable because of social disadvantage. Nor does it note the need for explicit attention to vulnerable social subgroups, for example, low-wage workers in prioritized occupational fields and low-income and minority pregnant women, infants, and toddlers. We are not questioning the rationality of defining major priority groups according to occupation or of using biological criteria to further prioritize within the general population. Rather, our concern is with the absence of attention to both biological and social risk factors, which must be addressed to overcome the many social barriers to equal opportunity for vaccination.

Well-documented evidence of existing healthcare disparities suggests that during a pandemic shortages of influenza vaccine, antiviral drugs, inpatient services, and healthcare staff will disproportionately affect persons in socially disadvantaged groups. To limit the crowds that might occur at hospitals and clinics, plans for the release of stockpiles of vaccines, medications, or both could include distribution from private pharmacies or doctors’ offices. However, because private pharmacies and private practitioners are less likely to be located in lower income neighborhoods, plans to make access to potentially lifesaving vaccines and drugs speedier and more equitable might, in fact, exacerbate disparities. Distribution plans may need to include mobile community health centers (staffed by nurses and nurse practitioners) that can travel to low-income areas, along with a variety of community medical and other service providers and nontraditional sites like soup kitchens, sheltered workshops, and transit points, which have become popular places for administering yearly influenza vaccine (*40*). Other factors, such as the availability of transportation to a hospital, might also become more important during a pandemic. Access to a private car may be a major determinant of who is able to obtain care, presenting constraints like those that led to disparities in evacuation from New Orleans before Hurricane Katrina. To ensure that disadvantaged communities are reached and that resources are equitably allocated during an influenza pandemic, preparedness plans can and should involve community-based providers and organizations that are familiar with vulnerable groups.

## Conclusions

Social group disparities in exposure, susceptibility, and access to timely and effective treatment for a variety of diseases have been well documented in the United States. Influenza pandemic preparedness plans that fail to explicitly provide guidelines on how to mitigate these issues could lead to decisions that may, on the surface, seem reasonable, but that are likely to exacerbate social group disparities in health outcomes. Given the existence of major disparities in health and healthcare, we cannot expect pandemic preparedness and response planning to eliminate the deep divides that exist between socioeconomic and racial/ethnic groups. These disparities can, however, be minimized through careful planning that considers and proactively addresses vulnerability at each level: exposure to disease, susceptibility to disease if exposed, and treatment of disease. Public officials should systematically consider the additional barriers faced by socially disadvantaged groups at each of these levels and then actively seek ways to address those barriers. Local service providers, leaders of community-based organizations and other organizations working with socially vulnerable groups, and leaders of labor unions representing low-wage service workers are likely to have valuable insights and should be included in the planning process. Plans calling for stakeholder involvement without explicitly emphasizing the need to involve representatives of socially disadvantaged groups are unlikely to be effective at minimizing social disparities during an influenza pandemic.

We have focused here on the United States, but similar fundamental principles—the need for systematic and concrete planning to minimize the social disparities that can be expected to occur in the face of natural disasters such as an influenza pandemic—apply worldwide. Countries with universal financial access to healthcare and strong social safety nets will be best positioned to minimize such disparities. Countries in which large proportions of the population are impoverished or otherwise socially excluded and countries that have more limited resources and weaker public health and social welfare infrastructures will face the greatest challenges. The framework used here—considering and proactively addressing social vulnerability in exposure to pathogens, susceptibility to disease once exposed, and consequences of illness—should be applicable across national and subnational settings.
